# Examining the Feasibility and Acceptability of Identifying Signs of Social Anxiety, in University Students Aged 18–25, While Watching 360° Immersive Films

**DOI:** 10.1111/inm.13499

**Published:** 2025-01-10

**Authors:** Jessica Eve Jackson, Ann Cox, Chris Windmill, Reza Safari

**Affiliations:** ^1^ Centre for Children and Young People's Research, School of Health Sciences University of Nottingham Nottingham UK; ^2^ Derbyshire Healthcare NHS Foundation Trust and Keele University Derby UK; ^3^ College of Science and Engineering University of Derby Derby UK; ^4^ College of Health, Psychology, Health and Social Care University of Derby Derby UK

**Keywords:** adolescents, heart rate monitoring, mental health, social anxiety disorder, virtual reality, young people

## Abstract

Timely, accurate assessment and treatment for social anxiety disorder (SAD) in young people is crucial. There is potential for the adoption of tailored virtual reality interventions for a complementary diagnostic tool using heart rate monitoring as a response indicator. This study examined the feasibility and acceptability of this concept by exposing healthy individuals, aged 18–25, to developed 360° immersive films while collecting heart rate sensor data. Two immersive scenarios were designed with a participatory advisory youth group. A clinical consultant nurse mapped scenario events against validated routine assessment subscales in child and adolescent services. The films were shown to seven participants through a VR headset while monitoring heart rate. They provided qualitative feedback on their quality, acceptability and usability. The study indicated that this approach has the potential to enhance assessments in child and adolescent mental health services. The study has several limitations which are discussed with recommendations for consideration if this concept is taken further. The intervention could act as a potential portable, user‐friendly complementary diagnostic tool for clinicians in practice. However, further research is needed to examine its validity.

## 1 Introduction

Social anxiety disorder (SAD) is described as a heightened fear of judgement of others to the point where a person actively avoids social situations such as public speaking, meeting new people or eating/drinking in public where potential scrutiny, humiliation or embarrassment may occur (American Psychiatric Association 2024a, 2024b). A global prevalence of young people who meet the threshold criteria for social anxiety is reported to be more than 1 in 3 (36%) (Jefferies and Ungar 2020). In England, referrals for Child and Adolescent Mental Health Services (CAMHS) in the National Health Service (NHS) have increased from 326 720 referrals in February 2021 to 450 355 referrals in December 2023 (NHS Digital 2023). This increase in demand for services means clinicians need to assess, diagnose and treat SAD effectively and efficiently.

The current National Institute of Health & Clinical Excellence (NICE 2013) guidance for diagnosing SAD in young people, recommends a series of subjective assessments through interviews with primary care and community workers before assessment with clinical specialists within CAMHS. This is supported by the collection of self and parent reports of validated routine outcome measures such as the Revised Child Anxiety and Depression Scale (RCADS) (Radez et al. 2021), which is validated for children aged 8–18 years, or the SPIN Social Phobia Inventory (Connor et al. 2000), which is validated for children aged 13 years and over. However, despite people with SAD presenting with strong physical symptoms, such as a rapid heart rate, nausea, sweating, or panic attacks (Szuhany and Simon 2022) they are not currently measured in the assessment process (NHS 2024).

With social anxiety being one of the most prevalent disorders for young people in England, ensuring a timely and correct diagnosis and developing the most appropriate treatment plan is important for a positive outcome (Hickling, Dabrowski, and Williams 2024). Misdiagnosis of social anxiety is commonplace and has been evidenced in practice and in the literature over recent years, with many young people being signposted for inappropriate autism assessments (Wittkopf et al. 2022). The effect of misdiagnosis can be that young people are offered inappropriate and ineffective treatment interventions, which subsequently impacts on recovery and longer time required within mental health services, or they are added to lengthy waiting lists with no or very little intervention.

Conducting an efficient assessment for an accurate diagnosis is important for the young person and the health economy. Another challenge in children's psychiatric services is that while there are diagnostic manuals that help inform clinicians if a diagnostic criterion has been met, the assessment and judgement is subjective by the clinician in assessing the child to have met a criterion (American Psychiatry Association 2024a, 2024b). There are many symptoms which are shared between mental health disorders and so misdiagnoses are commonplace across CAMHS (Aggarwal and Angus 2015; Bjønness, Grønnestad, and Storm 2020; O'Connor et al. 2020).

The use of virtual reality as part of an exposure intervention for SAD has been explored with positive outcomes (Chard and van Zalk 2022; Emmelkamp, Meyerbröker, and Morina 2020; Horigome et al. 2020). These interventions focus on treatment and intervention, rather than assessment and efficacy of diagnosis. In recent years, there have been technological developments for making more objective measurements, which were not previously available. Notably Parlatini et al. (2024) have developed an objective measurement of inattention, hyperactivity, and impulsivity to aid the efficacy of attention deficit hyperactivity disorder (ADHD) diagnosis with positive results. This enables professionals to use their clinical judgement alongside routine outcome measures and objective measurement to inform the diagnosis. Therefore, incorporating these technological developments as objective measurements of symptoms of social anxiety may also provide a more robust assessment and reduce the likelihood of misdiagnosis. This study aimed to examine the feasibility and acceptability of this concept by exposing healthy individuals, aged 18–25, to developed 360° immersive films while collecting heart rate sensor data. To assess the feasibility of the new diagnostic method, correlations are made between the RCADS/SPIN, routine analysis and the heart rate data. Besides the heart rate of participants was compared during and after each anxiety‐stimulating scenario within immersive intervention videos. For participant acceptability, the participants completed a qualitative evaluation questionnaire.

## 2 Method

### 2.1 Study Design

This is a cross‐sectional study with four key data collection points (1) online eligibility, (2) routine analysis, (3) immersive intervention and (4) evaluation questionnaire.

### 2.2 Ethical Procedures

The study obtained ethical approval from the researcher's higher intuition ethics committee before its implementation. Fully informed consent was obtained from all participants before taking part at each stage of the study: Online eligibility question, routine analysis and immersive intervention. Participants received a £10 Amazon voucher as a thank‐you for completing all three data collection points. Participants could withdraw their data from the study, up to 2 weeks, following the delivery of the interventions.

Participants were also monitored for common adverse side effects which have been linked to using VR equipment (Park and Lee 2020). For example, before watching the scenarios participants were informed to immediately take off the headset if they experienced motion sickness or physical discomfort.

### 2.3 Recruitment

Due to the study being a feasibility design, the sample size was decided based on the time and resources available for this project. University students were recruited through advertisements within communal spaces and services. Participants between the age of 18–25 years were invited to follow a QR code for online participant information. Participants were provided with contact details of the research team to ask further questions about the study. Participants were then invited to complete the online eligibility questionnaire.

### 2.4 Eligibility Questionnaire

The self‐reporting questionnaire was used to screen for inclusion/exclusion criteria. Participants who had previously received or were currently receiving (or in the process of being referred for) treatment for any mental health condition, cardiovascular or respiratory condition were excluded. We planned to assess the outcomes against the RACDS and SPIN assessment subscales as these were both aligned with the immersive intervention. We asked the younger participants to complete the RCADS scale because this scale is approved for use with the Child and Adolescent Mental Health Services. Older participants (> 19) were asked to complete the SPIN. This data (a) ensured that the participants included in this feasibility study were from a healthy population and was (b) included in the analysis of the intervention. Participants who did not meet the eligibility criteria were thanked for their interest and provided details of the higher institution's well‐being services.

### 2.5 Routine Assessment

The routine assessment was conducted by telephone with a CAMHS *Clinical Consultant Nurse*. A summary of their analysis was summarised qualitatively digitally. Firstly, all participants were asked to confirm their consent before being asked a set of questions that a clinician routinely uses to assess and explore a young person's symptoms. The rationale for undertaking this routine assessment was to imitate routine social anxiety diagnostic procedures and safeguard the participant's eligibility.

### 2.6 Immersive Intervention

The research team designed and developed two short scenario environments using 360° immersive technology. They mapped key events against the questions asked by RCADS and SPIN assessment subscales (see Table 1). The scripts for the scenarios were developed with young people from a youth participation advisory group to ensure that the immersive scenarios were appropriate and helpful for young people. They were each filmed and edited to be approximately 10 min in length.

**TABLE 1:** RCADS and SPIN assessment subscales questions.

SPIN questions	RCADS subscale questions
Q1. People in authority Q2. Blushing in front of others Q3. Parties/ social event Q4. Avoid talking to people I do not know Q5. Being criticised Q6. Fear of embarrassment Q7. Sweating Q8. Going to parties Q9. Centre of attention Q10. Talking to strangers Q11. Avoid giving speeches Q12. Avoid being criticised Q13. Heart palpitations Q14. Afraid of being watched Q15. Being embarrassed or looking stupid Q16. Avoid people in authority Q17. Trembling or shaking in front of others	Q1. I worry when I have done poorly at something Q2. I feel scared when I have to take a test Q3. I feel worried when I think someone is angry with me Q4. I worry that I have done badly with my schoolwork Q5. I worry I might look foolish Q6. I worry about making mistakes Q7. I worry about what people might think of me Q8. I feel afraid if I have to talk in front of my class Q9. I feel afraid if I make a fool of myself

The two scenarios were everyday experiences that all young people would encounter and included:

#### 2.6.1 Eating in Public

This scenario involved the participant sitting at a table and viewing a room of people eating various foods (sandwiches and crisps) and drinking beverages. Examples of specific events mapped against the subscales included a person spilling water, background talking and another being asked questions when a person was eating. Table 2 presents all mapped events of the *Eating in Public* scenario against the routine assessment scales.

**TABLE 2:** Mapped events of *Eating in Public* scenario.

Scenario event	Event SPIN ref.	Event RCADS ref.
Bottle dropped	6, 9, 14 and15	1, 5, 6, 7 and 9
Approached table	6, 9, 14 and 15	1, 5,6, 7 and 9
Person walked in the room	4, 10 and 14	5 and 7
Person joins the table	4, 6, 9, 10, 14 and 15	5, 7 and 8
Blushing in front of others	2 and 6	5 and 7
Asked a direct question	6, 9, 10 and 15	3, 5, 6 7 and 9
Asked a direct question (to the actor)	6, 9, 10 and 15	3, 5, 6, 7 and 9
Being offered crisps	4, 6, 9, 10 and 14	5, 6, 7, 8 and 9
Too many crisps in actor's mouth	2, 9, 14 and 15	1, 4, 5, 6, 7 and 9
Missed the bin	6, 9, 14 and 15	1, 5, 6, 7 and 9
Splashed water	6, 9,14 and 15	1, 5, 6, 7 and 9
Direct question	6, 9, 10 and 15	3, 5, 6, 7 and 9
Direct question	6, 9, 10 and 15	3, 5, 6, 7 and 9
Lots of people moving	3, 4, 9, 10, 14 and 15	1, 5, 6, 7 and9
Tripped on chair	2, 6, 9, 14 and 15	1, 5, 6, 7 and 9
Direct question	6, 9, 10 and 15	3, 5, 6, 7 and 9
Direct question	6, 9, 10 and 15	3, 5, 6, 7 and 9

#### 2.6.2 Giving a Presentation to a Class

This scenario involved the participant sitting in a classroom setting with other peers. There was one teacher at the front of the class who invited people in the class to present their work one at a time. Examples of specific events mapped against the subscale included different responses from the class and presentation styles. Table 3 presents all mapped events of the *Classroom* scenario against the routine assessment scales.

**TABLE 3:** Mapped events of *Classroom* scenario.

Scenario event	Event SPIN ref.	Event RCADS ref.
Asking how to display a picture	6, 14 and 15	1, 2, 3, 4, 5,6, 7, 8 and 9
Pupil speaks to another	5, 9, 11, 14 and 15	1, 3, 4, 5, 6, 7, 9
Teacher tells people not to talk	1, 5, 6, 9, 14, 15 and 16	1, 3, 4, 5, 6, 7, 8 and 9
Pupil picks up and plays with a phone	12, 14 and 15	1, 3, 4, 5, 6, 7, 9
Teacher tells the pupil to get off phone	1, 9, 12, 15 and 16	3, 4, 5, 6, 7, 9
Direct question	5, 6, 9, 10, 12, 14 and 15	1, 2, 3, 4, 5, 6, 7, 8 and 9
Open question	6, 9, 10 and 15	1, 2, 3, 4, 5, 6, 7, 8 and 9
Teacher feedback on the presentation	1, 5, 6, 9, 11, 14, 15 and 16	1, 2, 3, 4, 5, 6, 7, 8 and 9
Round of applause	9, 14 and 15	5, 7, 8 and 9
Requesting pupil to present who refuses	1, 5, 6, 9, 11, 12, 14, 15 and 16	1, 2, 3, 4, 5, 6, 7, 8 and 9
Pupil says excuse me to camera	4 and 9	3, 7 and 9
Pupil trips over desk	2, 6, 9, 14 and 15	5, 6, 7, 8 and 9
Phone goes off	6, 9, 11, 12 and 15	4, 5, 6, 7, 8 and 9
Struggling to pronounce words	5, 6, 11, 12, 14 and 15	1, 2, 3, 4, 5, 6, 7, 8 and 9
Pupil laughs and teacher addresses	1, 5, 9, 11, 14 and 15	1, 2, 3, 4, 5, 6, 7, 8 and 9
Pupil laughing in the background	5, 9, 11, 14 and 15	1, 3, 4, 5, 6, 7, 9
Pupil taps on the desk	5, 9, 12 and 15	1, 3, 4, 5, 6, 7, 8 and 9
Pupil laughing at the presenter	5, 9, 11, 14 and 15	1, 2, 3, 4, 5, 6, 7, 8 and 9
Pupil speaks to another pupil	5, 9, 11, 14 and 15	1, 3, 4, 5, 6, 7, 9
Direct question from the teacher	1, 5, 6, 9, 10, 12, 15 and 16	1, 2, 3, 4, 5, 6, 7, 8 and 9
Pupil laughing walking out	5, 6, 9, 12, 14 and 15	1, 2, 3, 4, 5, 6, 7, 8 and 9
Direct questions to the presenter	6, 9, 10 and 15	1, 2, 3, 4, 5, 6, 7, 8 and 9
Direct question presenter and feedback	1, 5, 6, 9, 10, 12, 15 and 16	1, 2, 3, 4, 5, 6, 7, 8 and 9

The films were played through virtual reality headsets (Meta Quest 2). This equipment has been used for clinical validation pilot studies and is highlighted as user‐friendly (Trinidad‐Fernández et al. 2023). The device is lightweight and portable and easy for the participant to wear. The Polar H10 *Heart Rate Monitor* was used to measure heart rate, which was self‐assembled by the participants around their chest. This device has accurate heart rate tracking outcomes in research (Schaffarczyk et al. 2022). Participants were sitting in a chair for the duration of the intervention. While watching the films the participant's view was recorded on a smart device. This enabled an observation of whether they were directly looking at the mapped event at the time it occurred. The data was downloaded and converted to a Microsoft Excel document.

### 2.7 Intervention Evaluation Questionnaire

The paper copy intervention evaluation questionnaire was administered immediately following participation. asked three qualitative questions to assess the practicalities of using the equipment: (1) What was your experience using the equipment? (2) Were there any challenges in using the equipment? (3) Was there anything you thought was positive in using the equipment? Participants were also asked their thoughts on each scenario.

### 2.8 Data Analysis

A Spearman correlation test was performed, to assess the correlation between the SPIN and RCADS scores with participants' heart rates. The total score of the scales was compared against the average heart rate during the entire immersive film. Then, the correlation between the heart rate after each event was assessed against the corresponding SPIN or RCADS scale items. We examined the impact of immersive films on HR using a paired *t*‐test. This compared the average baseline HR with HR while watching the films and the average HR during each event with the HR 10 s after each event. A reflexive coding analysis was adopted for the intervention evaluation questionnaire. The data were organised in Excel for exploration. The authors immersed themselves in the data, assigning labels to related themes. This qualitative data contribute insights to the usability and acceptability of the assessment approach beyond that obtained in the quantitative methods.

## 3 Results

**FIGURE 1:**
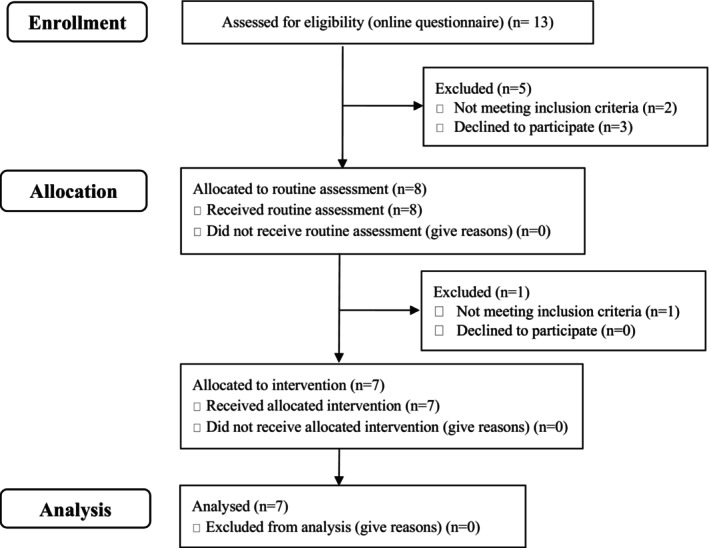
Flow diagram of included participants.

Figure 1 presents the participant flow diagram. 13 participants expressed an interest and were assessed for eligibility, two were excluded for not meeting the inclusion criteria and three declined to participate. This meant eight participants were allocated to the routine assessment (data collection point one). Following this, one participant was assessed at the high end of the scale for SAD and therefore was excluded from receiving the intervention. A total of seven participants received the intervention and are included in the analysis.

### 3.1 Participants Characteristic

Table 4 presents the participant characteristics and the outcome of the SPIN/RACDS scale and routine analysis.

**TABLE 4:** Participant information.

Gender	Ethnicity	Age	Scale	Score	Description	Routine analysis	Average HR
Male	Black British	21	SPIN	31	Moderate	Low	79
Female	Black African	25	SPIN	3	Low	Low	86
Female	White, British	21	SPIN	41	Moderate	Low/High on occasions	94
Female	Black British	23	SPIN	6	None/Mild	Low	91
Female	White British	19	RCADS	7	Mild	Low	100
Transgender	White British	22	SPIN	37	Moderate	Moderate	83
Female	White British	18	RCADS	4	Mild	Low	91

### 3.2 Analysis of Heart Rate Data

The results of the Spearman test did not show any correlation between the average heart rate and total SPIN scores nor for the heart rate during the events and the score of individual SPIN questions corresponding to these events within the immersive videos. Results are not presented. A correlation test was not conducted for the RCADS scores, as only two participants completed this questionnaire. There were no significant differences between the average baseline HR and the HR during the video. However, the HR after the events was significantly higher than during the events, Table 5.

**TABLE 5:** Heart rate, mean, standard deviation and *p* value.

Heart rate	Mean	SD	Mean dif.	SD	*p*
Baseline Café	87.60	8.65	0.05	3.64	0.967
Average Café	87.65	7.81			
Baseline classroom	88.68	9.42	0.98	3.80	0.553
Average classroom	89.68	7.58			
During café events	85.14	7.40	1.91	0.91	0.001
10 s after Café events	87.05	7.59			
During classroom events	88.46	8.71	1.78	1.14	0.012
10 s after classroom events	90.24	9.49			

**FIGURE 2:**
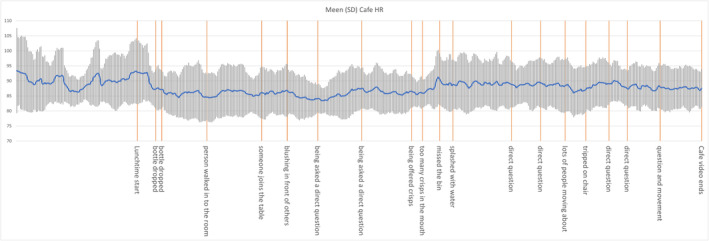
Participants HR during eating in public mean (blue line). The orange lines indicate the anxiety‐stimulating events during the immersive video.

**FIGURE 3:**
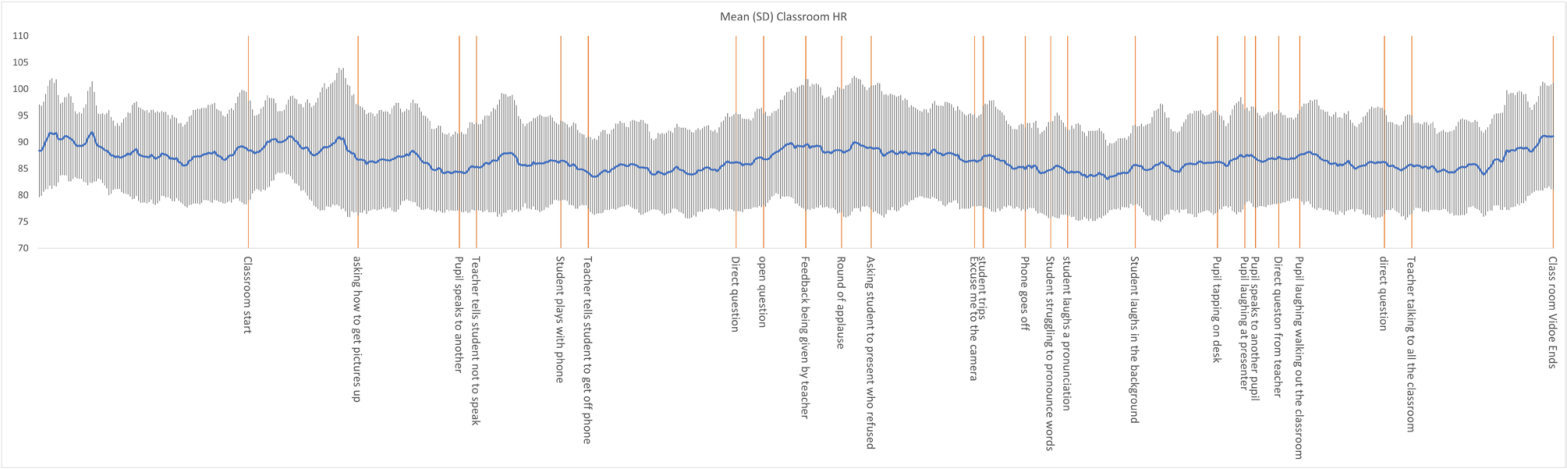
Participants HR during classroom; mean (blue line). The orange lines indicate the anxiety‐stimulating events during the immersive video.

Figures 2 and 3 are the HR (blue line) for two example participants during the films which identify peaks after some of the events. The orange lines indicate the anxiety‐stimulating events during the immersive video. Figure 2 indicates that upon starting the stimulus the participant's HR peaked at their highest values before dropping to a value significantly below their average resting heart rate. After they are directly asked a question, breaking the observer/participant barrier their heart rate rises for the remainder of the experiment with localised peaks occurring where the viewer is directly involved in the activity. Figure 3 indicates significantly more variation in the participant's HR with three clear patterns: normal classroom activity shows little in the way of HR variation; normal negative/corrective behaviour shows a peak with the HR dropping back towards normal once the teacher applies corrective behaviour; and on‐going events where the participant's HR rises significantly for the duration until one of the other two classes of behaviour occur.

### 3.3 Participants Perception

Due to the small sample size analysis of the effect cannot be made for participants' direct view of the events as they occurred in the films. However, we can report that participants looked directly at the *Eating in Public* events 84% of the time when it first occurred and 12% of the time during its occurrence (see Table 6).

**TABLE 6:** *Eating in Public*: Participant assessment of direct view during each event.

*Eating in Public* events	Participant assessment of direct view during each event						
	P1	P2	P3	P4	P5	P6	P7
Bottle dropped	Yes	Yes	Yes	Yes	During	Missing	Missing
2 Approached table	During	Yes	Yes	Yes	No	Missing	
3 Person walked in the room	During	Yes	Yes	Yes	Yes	Yes	
4 Person joins the table	Yes	Yes	Yes	Yes	Yes	Yes	
5 Blushing in front of others	Yes	Yes	Yes	Yes	Yes	Yes	
6 Asked a direct question	Yes	Yes	During	Yes	Yes	Yes	
7 Asked a direct question (to the actor)	Yes	During	Yes	Yes	Yes	Yes	
8 Being offered crisps	Yes	Yes	Yes	Yes	Yes	Yes	
9 Too many crisps in actor's mouth	During	During	Yes	Yes	Yes	Yes	
10 Missed the bin	Yes	Yes	Yes	Yes	Yes	Yes	
11 Splashed water	During	Yes	Yes	During	Yes	Yes	
12 Direct question	During	During	Yes	Yes	Yes	Yes	
13 Direct question	Yes	Yes	Yes	Yes	During	Yes	
14 Lots of people moving	Yes	Yes	Yes	Yes	Yes	Yes	
15 Tripped on chair	During	Yes	Yes	Yes	Yes	Yes	
16 Direct question	Yes	Yes	Yes	Yes	Yes	Yes	
17 Direct question	Yes	Yes	Yes	Yes	Yes	Yes	

In the classroom event, participants looked directly at the events when they first occurred only 44% of the time and directly at it during the event 17% of the time (see Table 7). Due to technical error, recording was not achieved for a participant in each of the scenarios.

**TABLE 7:** *Classroom*: Participant assessment of direct view during each event.

Classroom events	Participant assessment of direct view during each event						
	P1	P2	P3	P4	P5	P6	P7
Asking how to display a picture	Missing	Yes	Yes	Missing	Yes	During	Yes
2 Pupil speaks to another		No	During	Missing	No	No	No
3 Teacher tells people not to talk		During	Yes	Missing	Yes	Yes	During
4 Pupil picks up and plays with a phone		No	Yes	No	No	Yes	Yes
5 Teacher tells the pupil to get off phone		No	No	No	No	Yes	During
6 Direct question		No	Yes	Yes	Yes	Yes	Yes
7 Open Question		During	During	No	During	Yes	No
8 Teacher feedback on the presentation		No	During	During	Yes	Yes	Yes
9 Round of applause		Yes	Yes	During	No	Yes	Yes
10 Requesting pupil to present who refuses		Yes	Yes	During	Yes	Yes	Yes
11 Pupil says excuse me to camera		Yes	Yes	During	During	Yes	Yes
12 Pupil trips over desk		Yes	Yes	Yes	Yes	Yes	Yes
13 Phone goes off		No	Yes	No	Yes	During	Yes
14 Struggling to pronounce words		Yes	Yes	Yes	Yes	Yes	Yes
15 Pupil laughs and teacher addresses		No	During	No	Yes	Yes	During
16 Pupil laughing in the background		No	No	No	No	Yes	No
17 Pupil taps on the desk		No	During	No	During	Yes	No
18 Pupil laughing at the presenter		No	No	No	No	Yes	No
19 Pupil speaks to another pupil		No	No	No	No	Yes	During
20 Direct question from the teacher		During	During	During	Yes	Yes	During
21 Pupil laughing walking out		Yes	Yes	Yes	Yes	Yes	Yes
22 Direct questions to the presenter		Yes	Yes	Yes	Yes	Yes	Yes
23 Direct question presenter and feedback		During	During	Yes	Yes	Yes	During

### 3.4 Evaluation Questionnaire

The qualitative feedback on the usability and acceptability of the assessment approach indicated that participants felt the goggles provided an immersive experience with spatial audio, making participants feel engaged and part of the scene, though one noted the actors should be younger for CAMHS realism. The motion and interaction within the scenes were somewhat limited, and balancing independent agency for exploration with directly looking at the scenario events posed a challenge and led to some uncertainty about where to focus. The headset was comfortable despite several participants needing to make minor adjustments. No discomfort from prolonged use was reported. However, participants also reported several issues which slightly disrupted the immersive experience. These included image freezing, tunnel vision effect and visible parts of the camera rig.

## 4 Discussion

This study set out to pilot two 360° immersive films developed to be mapped against validated measurement scales for SAD and in consultation with an advisory participatory youth group. It examined the feasibility and acceptability of playing these immersive films through a virtual reality headset while monitoring heart rate in a healthy population as a potential diagnostic tool. Due to a small sample size, only two cases for RCADS and five cases for SPIN data a statistical analysis of these data was not performed as it would not be valid. In hindsight, although the scenarios were mapped against both RACDS and SPIN, using the SPIN only for these included participants would have been a better data collection approach. Additionally, there was not significant difference in the baseline HR and the HR while watching the films. However, the results have indicated a trend in increased HR of individual participant responses to the recorded events. This is an interesting finding, indicating that immersive videos may be used to elicit change in HR and, therefore, an indicator of anxiety.

There were several challenges which limits this study and would need consideration in any further research exploring this assessment approach. For example, this was a healthy population that did not meet the threshold for SAD. Another challenge with this data set is that we cannot determine whether participants were relaxed at the start of the videos and therefore, we do not have accurate baseline data. We did begin recording heart rates during the intervention brief to ensure the participants became accustomed to the environment. It might be possible that their heart rate was elevated in the anticipation of watching the films and therefore we cannot evidence it as an accurate baseline measure. A rest to control the baseline physiological variables is a common shortcoming highlighted by Catai et al. (2020) who further recommend asking participants not to talk, sleep or move during the collection. Therefore, this means that we cannot comment on the validity of this approach and further research with a larger sample is needed. However, in developing and filming the scenarios and exploring the feasibility of adopting this approach as a clinical assessment, we have identified several insights which have important implications for further exploration.

Overall, the intervention evaluation highlighted that wearing the goggles provided a highly immersive experience. One participant stated that using the goggles allowed them to watch the scenarios free from other distractions in the room. Another commented on the spatial audio, indicating that it added another layer of immersion by delivering sound from all directions. Audio immersion was also recognised in a qualitative investigation of user experiences of immersive films (Williams, Shepstone, and Murphy 2022). In this study, the authors suggest measuring biomarkers, such as heart rate, to examine the absorption of auditory immersion. The decision to use 360° content was to deliver a dynamic and interactive viewing experience. One participant did indicate they felt part of the scene which enhanced their engagement. This feedback indicates participants thought both scenarios were realistic, although one participant stated that the actors should have been younger for service users of CAMHS. Wider literature also recognises the unique actor–audience relationship needed for effective engagement in immersive films (Gallon 2021). For practical reasons, this was not possible in this study, however, this is crucial in any further development of such a scenario for this target group.

The scenarios were filmed using one camera, which avoided the need to stitch together footage from multiple cameras and risk creating visible streams. The participants were positioned within the scenario as an additional person. This meant they remained static as a passive viewer of the environment with actors interacting with each other around them. Although there were moments where an actor looked directly at the camera as if looking at the participant, the realistic motion and interaction of people within the scene were limited. This means that there was a challenge in balancing between allowing the participants the independence to explore the scene while exposing them to the series of mapped events. For example, in the recording of the participant's view, there appeared to be incidents where they may have felt unsure of where to focus their attention, particularly in the classroom scenario. Therefore, if there is limited directional guidance it may lead to participants feeling disoriented and can make it harder to follow and maintain immersion (Masia et al. 2021).

The weight and fit of the headset were reported as comfortable for all participants, despite having to adjust it between scenarios as it had slipped forward slightly. Participants did not report any discomfort linked to extended use of VR goggles such as eye strain, headaches, and neck pain from using the equipment (Arif, Khan, and Khan 2021). This indicated that the duration and pace of the scenarios were appropriate to safeguard against this. However, there were several technical issues which materialised during data collection. For example, one participant stated that when they were watching one of the scenarios, the image froze twice which was distracting. These scenarios were being streamed from an online server and this frozen image may have been caused by a loss of connection for a short period. Delivering high‐quality 360° content is challenging because it requires significantly more data, resulting in large file sizes that can be difficult to stream or download on slower internet connections (Bouraqia et al. 2020). These issues can disrupt the viewing experience and may require technical knowledge to resolve. One participant also commented on a tunnel vision effect that detracts from the immersive experience. This was due to the 360° filming creating a slightly distorted perspective, especially at the edges of the field of view (Chakareski et al. 2022). Additionally, parts of the camera rig were inadvertently visible in the footage if the participant turned vertically, breaking the illusion of being present in the scene. This is a limitation in the technology and needs further consideration for future research in this area. Despite this, the approach shows potential for user customisation which can make the viewing experience more enjoyable and tailored to personal preferences. Further development should also require careful consideration of the needs of a more diverse user population, equitable language and cultural experiences and ensuring the approach is accessible (Dudley et al. 2023).

## 5 Conclusion

This study has indicated that identifying signs of social anxiety through watching 360° immersive films and measuring heart rate is feasibly viable to enhance assessments for young people in mental health services. However, there are several lessons through the development of the scenarios, which need to be considered if this concept is taken further. To achieve high‐quality, realistic 360° content requires the right resources and expertise.

## 6 Relevance to Clinical Practice

This study has shown that the virtual reality equipment is easy and comfortable for people to use. Developing evidence‐based tailored scenarios for patients to watch while recording their heart rate may help professionals identify social anxiety quickly and accurately. The intervention could serve as a potential portable, user‐friendly complementary diagnostic tool for clinicians. However, further research is necessary to confirm its validity and effectiveness in clinical practice.

## Author Contributions

J.E.J.: conceptualisation, data curation, investigation, methodology, project administration, formal analysis, writing the original, review and editing. A.C.: conceptualisation, investigation, formal analysis, writing the original, review and editing. C.W.: conceptualisation, review and editing. R.S.: conceptualisation, methodology, formal analysis, writing the original, review and editing.

## Ethics Statement

The study obtained full ethical approval from the College of Health, Psychology and Social Care Research Committee.

## Consent

Fully informed consent was obtained from all participants who took part in this study.

## Conflicts of Interest

The authors declare no conflicts of interest.

## Data Availability

The data sets used and/or analysed during the current study are available from the corresponding author upon reasonable request.
